# Publication rate of abstracts presented at Japan Geriatrics Society Annual Meetings (2011–2012): a retrospective observational study

**DOI:** 10.1186/s13104-018-3156-5

**Published:** 2018-01-16

**Authors:** Junpei Komagamine, Masaki Kobayashi

**Affiliations:** Department of Internal Medicine, National Hospital Organization Tochigi Medical Center, 1-10-37, Nakatomatsuri, Utsunomiya, Tochigi 3208580 Japan

**Keywords:** Annual meetings, Abstracts, Conference, Geriatrics, Publication

## Abstract

**Objective:**

We aimed to determine the publication rate of abstracts presented at Japan Geriatrics Society Annual Meetings. Publication rates were determined by searching for full-text publications up to September 2017 in the MEDLINE database. Factors associated with publication were determined.

**Results:**

In total, 618 abstracts presented at Japan Geriatrics Society Annual Meetings (2011–2012) were included. Of those, 146 (23.6% [95% CI 20.3–27.0%]) were published in peer-reviewed journals indexed in MEDLINE. The median time to publication was 13.0 months (interquartile range 6.0–25.8 months). More than 90% were published within 4 years. The publications appeared in 64 different journals, and 87.0% were published in English-language journals. Multivariable analysis revealed more frequent publication of oral presentations (25.4% vs 16.9% of poster presentations; adjusted OR 1.79 [95% CI 1.05–3.06]), randomized controlled trials (66.7% vs 22.8% for other study designs; adjusted OR 10.79 [95% CI 3.02–38.53]) and studies with n ≥ 100 (28.7% vs 18.4% of studies with n < 100; adjusted OR 1.97 [95% CI 1.32–2.95]). Because more than three-fourths of the abstracts presented at Japan Geriatrics Society Annual Meetings remained unpublished within 5 years after the conferences, additional efforts may be needed to promote their publication.

## Introduction

A scientific presentation at an annual meeting is considered as an initial method to share novel research findings before their publication in a peer-reviewed journal [1–3]. However, a previous systematic review of 79 studies reported that the average publication rate of abstracts presented at the annual meetings for many specialties was 44.5% [4]. For recent 19 studies published from 2014 to 2016, the average publication rate was 41.5% [5]. Thus, it is estimated that more than half of all abstracts presented at annual meetings are not published after the conference. Therefore, some experts have proposed using a scientific meeting’s abstract-to-publication ratio as a quality indicator of its scientific value [1, 6–10].

To our knowledge, no studies have ever been conducted to determine the publication rate of abstracts presented at the annual scientific meetings for geriatric medicine [4, 5]. Furthermore, few studies have evaluated this outcome for the Japanese medical specialty meetings [2]. Therefore, we conducted this study to determine the publication rate and factors associated with publication of abstracts presented at Japan Geriatrics Society Annual Meetings.

## Main text

### Methods

A retrospective observational study of abstracts presented at the 2011 and 2012 Japan Geriatrics Society Annual Meetings was conducted. The aims of this study were to determine the publication rate of abstracts presented at Japan Geriatrics Society Annual Meetings and to identify factors associated with publication. These meetings were chosen to allow a sufficient time to publication, as more than 95% of published articles are published within 5 years of initial presentation [4]. All 630 abstracts from these meetings were reviewed. Twelve were excluded (3 withdrawn and 9 published more than 6 months before the index annual meetings). The following information was extracted: year of the annual meeting, format, study design, number of authors, affiliation of authors, and sample size of the study.

To evaluate the primary outcome, a publication search was conducted by entering author names as keywords in the MEDLINE database to examine publications from 1946 to September 2017. This search began on October 2, 2017, and was completed on November 2, 2017. Only publications published up to September 2017 were included. Because the deadline for abstract submission to the Japan Geriatrics Society Annual Meeting is approximately 6 months before the conference, publications published more than 6 months before the conference were excluded. A prior study reported good inter-observer reliability for searching publications [11], and the number of abstracts searched was not overly substantial. Therefore, a single investigator (J.K.) performed this search. The name of the first author was used as a keyword to search for the article. If the initial search identified no publications corresponding to the presented abstracts, the name of the second author was used in a search. Abstracts were considered published if a matching full-length article was identified using this search strategy. Based on a previous study, brief reports and research letters were also considered published articles because they are subject to peer review and indexed in MEDLINE [1]. Retrieved publications were compared with the corresponding abstract to ensure that they represented the same work. Only published articles that were nearly identical in terms of target population, hypothesis and study design were judged to be the same work. Articles that included some of the data presented in the abstract (e.g., a smaller cohort) were also regarded as the same work [12]. However, the decision regarding whether the identified article represented the same work as the abstract presented at the annual meeting was difficult for 10 abstracts; these abstracts were discussed, and the judgments resolved by consensus among two authors (J.K. and M.K.). Abstracts were considered unpublished if this search strategy was unable to obtain matching results. Authors of abstracts were not contacted to elicit whether the research had been published in peer-reviewed journals. For the identified articles, information on the name of the journal and the publication date were retrieved.

The sample size was determined based on prior similar studies. Two annual meetings were chosen to include more than 400 abstracts because the mean number of abstracts in prior studies was approximately 400 [4]. Descriptive statistics were used for reporting the results. The primary outcome was calculated as the percentage of all included abstracts that were published in peer-reviewed journals. The 95% confidence interval (CI) was also determined for this outcome. For published abstracts, the median time (months) from the annual meeting to publication was calculated. The accumulated number and the proportion of abstracts published every 6 months were also determined. In addition, information on peer-reviewed journals in which the abstracts were published was presented using descriptive statistics. The associations between publication and the following variables were evaluated by univariate analysis using binary logistic regression: year of the annual meeting (2011 or 2012), format (oral or poster), study design (randomized controlled trial or other), number of authors (n < 3 or n ≥ 3) [13], affiliation of authors (university-associated or non-university-associated), and sample size of the study (n < 100 or n ≥ 100) [14]. A multivariate analysis was also conducted using these variables. These analyses were conducted using Stata version 15 (LightStone, Tokyo, Japan), and the level of statistical significance was p < 0.05.

### Results

A total of 618 abstracts (297 in 2011 and 321 in 2012) were evaluated. Of these, 488 (79.0%) were oral presentations, and 130 (21.0%) were poster presentations. The median number of authors was 5.0 (interquartile range 3.0–7.0). Of all abstracts, 146 (23.6% [95% CI 20.3–27.0%]) were published in a peer-reviewed journal indexed in MEDLINE. The median time to publication was 13.0 months (interquartile range 6.0–25.8 months). Approximately 70% were published within 2 years, and more than 90% were published within 4 years (Table 1).

**Table 1:** Distribution of 146 published abstracts by the time of presentation at the 2011 and 2012 Japan Geriatrics Society Annual Meetings to publication

Time from annual meeting presentation to publication	Year of conference^a^		Accumulated number of published abstracts^a^, n (%)
	2011	2012	
Before the conference^b^	7	9	16 (11.0)
0–5 months	7	13	36 (24.7)
6–11 months	17	12	65 (44.5)
12–17 months	14	10	89 (61.0)
18–23 months	7	6	102 (69.9)
24–29 months	7	6	115 (78.8)
30–35 months	4	6	125 (85.6)
36–41 months	4	2	131 (89.7)
42–47 months	3	4	138 (94.5)
48–53 months	3	1	142 (97.3)
54–59 months	1	3	146 (100.0)
After 60 months	0	0	146 (100.0)

^a^Values are the number of published abstracts, with the percentage of the total number of published abstracts in parentheses

^b^Within 6 months before the annual meeting

Table 2 shows the characteristics of the abstracts that were associated with publication. In the univariate analysis using binary logistic regression, publication was statistically significantly more frequent for oral presentations (25.4% vs 16.9% for poster presentations; OR 1.67 [95% CI 1.01–2.76]), randomized controlled trials (66.7% vs 22.8% for other designs; OR 6.78 [95% CI 2.01–22.86]), and studies with a larger sample size (28.7% for n ≥ 100 vs 18.4% for n < 100; OR 1.78 [95% CI 1.22–2.61]). Based on multivariate analysis adjusted for six selected variables, oral presentation, randomized controlled trial and larger sample size were the only independent predictive factors to be statistically significantly associated with publication.

**Table 2:** Characteristics associated with publication of abstracts presented at the 2011 and 2012 Japan Geriatrics Society Annual Meetings

Characteristics	Total^a^	Number (%) of published abstracts^a^	Odd ratio (95% confidence interval)^b^	
			Univariate analysis	Multivariate analysis^c^
Year of the conference				
2011	297	74 (24.9)	1 [reference]	1 [reference]
2012	321	72 (22.4)	0.87 (0.60–1.26)	0.88 (0.60–1.30)
Presentation format				
Poster	130	22 (16.9)	1 [reference]	1 [reference]
Oral	488	124 (25.4)	1.67 (1.01–2.76)*	1.79 (1.05–3.06)*
Study design				
Other^d^	606	138 (22.8)	1 [reference]	1 [reference]
Randomized controlled trial	12	8 (66.7)	6.78 (2.01–22.86)*	10.79 (3.02–38.53)**
Sample size^e^				
< 100	299	55 (18.4)	1 [reference]	1 [reference]
≥ 100	307	88 (28.7)	1.78 (1.22–2.61)*	1.97 (1.32–2.95)*
Number of authors				
< 3	115	24 (20.9)	1 [reference]	1 [reference]
≥ 3	503	122 (24.3)	1.21 (0.74–1.99)	0.98 (0.56–1.72)
Affiliation of authors				
Non-university-associated	162	30 (18.5)	1 [reference]	1 [reference]
University-associated	456	116 (25.4)	1.50 (0.96–2.35)	1.44 (0.88–2.35)

^a^Values are the number of abstracts, with the percentage of the total number of published abstracts according to subgroups classified by each variable in parentheses

^b^The level of statistical significance was set at p < 0.05. Asterisks indicate a significant association between the selected variables and publication; * *p* < 0.05, ** *p* < 0.001

^c^Adjusted for the year of the conference, format, study design, number of authors, affiliation of authors, and sample size of the study

^d^These include observational studies, case reports, case series, simulation analyses, and non-human studies

^e^Twelve abstracts in which the sample size was not documented were excluded

Table 3 shows the journals in which the abstracts were published. In total, 146 abstracts presented at the 2011 and 2012 Japan Geriatrics Society Annual Meetings were published in 64 different journals (61 English-language journals and three Japanese-language journals). Of those, 127 abstracts (87.0%) were published in English-language journals.

**Table 3:** List of journals in which the 146 abstracts presented at the 2011 and 2012 Japan Geriatrics Society Annual Meetings were published

Journal	Number of publications^a^ (n = 146)
*Geriatr Gerontol Int*	38 (26.0)
*Nihon Ronen Igakkai Zasshi* [in Japanese]	17 (11.6)
*J Am Geriatr Soc*	8 (5.5)
*PLoS One*	4 (2.7)
*Arch Gerontol Geriatr*	3 (2.1)
*Hiroshima J Med Sci*	3 (2.1)
*J Am Med Dir Assoc*	3 (2.1)
*Hypertens Res*	3 (2.1)
*Behav Brain Res*	2 (1.4)
*BMJ Open*	2 (1.4)
*Br J Nutr*	2 (1.4)
*Cardiovasc Diabetol*	2 (1.4)
*Intern Med*	2 (1.4)
*J Atheroscler Thromb*	2 (1.4)
*J Bone Miner Res*	2 (1.4)
*J Hypertens*	2 (1.4)
*J Neurol Sci*	2 (1.4)
*J Nippon Med Sch*	2 (1.4)
*Tokai J Exp Clin Med*	2 (1.4)
Others^b^	45 (30.8)

^a^Values are the number of published abstracts, with the percentage of total publications in parentheses

^b^These consisted of 45 journals (two Japanese-language journals and 43 English-language journals)

### Discussion

This study showed that the overall publication rate of abstracts presented at Japan Geriatrics Society Annual Meetings was 23.6%. More than 90% of all published abstracts were published within 4 years after the conference. Publication was significantly more frequent for oral presentations, randomized controlled trials, and studies with a larger sample size.

To our knowledge, this study is the first to determine the publication rate of abstracts presented at annual scientific meetings for geriatric medicine. The publication rate in the present study was lower than the publication rate in recent studies and a previous systematic review with regard to other specialties outside Japan [1, 4–10] as well as the only Japanese study for the Annual Meeting of the Japanese Orthopaedic Association [2]. Given that author origin (from non-English-language countries vs. from English-language countries) might affect the abstract publication rate [4, 9, 15], our findings might be limited to Japan and might not be generalizable to authors originating from English-language countries. However, a past systematic review reported an effect of specialties on the publication rate of abstracts [16]. Therefore, it is unclear whether the lower publication rate observed in this study is attributable to the country (Japan), the specialty (geriatrics), or other factors. Further studies of the publication rates of abstracts presented at annual meetings for geriatric medicine in other countries are needed to evaluate the external validity of our findings.

In this study, six variables (year of the annual meeting, format, study design, number of authors, affiliation of authors, and sample size of the study) were evaluated to predict the occurrence of publication. Our findings support the past systematic reviews and recent studies in that oral presentations were more likely to be published than poster presentations [4, 5, 10, 15–18]. However, it is unknown why oral presentations are more likely to be published than poster presentations [15], although it is anecdotally noted that a higher-quality study might be selected as an oral presentation by the programme committee [15, 19]. Our results were also consistent with those of previous studies in showing that randomized controlled trials were more likely to be published than studies with other designs [1, 4]. These findings may reflect the emphasis that randomized controlled trials are the gold standard study design for examining the efficacy of treatments [1]. Consistent with a recent study [14], our study showed that a larger sample size (n ≥ 100) was significantly associated with more frequent publication. However, given that prior studies have shown conflicting results for the effect of sample size on the likelihood of publication [1, 4, 20], further studies are needed to evaluate this association.

Prior studies reported that the most frequently cited barriers to abstract publication are lack of time and lack of interest [21–24]. However, it is unclear whether these factors are also barriers to publication among Japanese investigators because all prior studies were conducted outside Japan. Given that more than three-fourths of abstracts presented at Japan Geriatrics Society Annual Meetings were unpublished within 5 years after the conferences, further studies to determine the barriers to publication among Japanese investigators and additional efforts to increase the publication rate of abstracts presented at Japan Geriatrics Society Annual Meetings are needed.

### Conclusions

The publication rate of abstracts presented at Japan Geriatrics Society Annual Meetings was 23.6%. Further studies of scientific meetings for geriatric medicine in other countries are needed to evaluate the external validity of our findings. Because the publication rate of these Japan Geriatrics Society Annual Meetings was lower than the average rate for all previously studied scientific conferences, additional efforts may be needed to increase the rate of publication of abstracts presented at these annual meetings.

## Limitations

Several limitations must be mentioned. First, publication status was determined based on a single database. Furthermore, we did not contact any authors of the abstracts to obtain information about publication. Second, abstracts from only two meetings were included. Furthermore, we evaluated the publication rate of abstracts only for the Japan Geriatrics Society Annual Meetings. Third, factors that might be associated with publication, such as abstract results [4] and abstract quality [25], were not evaluated. Fourth, a prior study reported that there were often various inconsistencies between abstracts presented at annual meetings and matching published articles [26], and for some abstracts included in this study, judging whether they were the same work as the identified articles was difficult. Finally, the possibility of duplicate presentations of the same study at multiple meetings [27] was not investigated.

## Abbreviations

CI: confidence interval
OR: odds ratio
